# The Caribbean enigma: the presence of unusual cryptic diversity in intertidal mites (Arachnida, Acari, Oribatida)

**DOI:** 10.1007/s13127-019-00416-0

**Published:** 2019-09-04

**Authors:** Tobias Pfingstl, Julia Baumann, Andrea Lienhard

**Affiliations:** Institute of Biology, Department for Biodiversity and Evolution, University of Graz, Universitaetsplatz 2, 8010 Graz, Austria

**Keywords:** *Carinozetes*, Selenoribatidae, Biogeography, Morphometry, Cytochrome oxidase I

## Abstract

The definition, as well as the existence of cryptic species, is still a subject of controversial debates. Some scientists claim that cryptic diversity is a real phenomenon that should be extensively studied while others argue that cryptic species do not exist as they are nothing more than an incompatibility of species concepts. We investigated the enigmatic case of two widely distributed Caribbean intertidal oribatid mites, *Carinozetes bermudensis* and *Carinozetes mangrovi*, consisting of five distinct genetic lineages. Morphological features allowing to clearly distinguish between these lineages are absent, and despite certain congruence with genetic data, comprehensive morphometric analyses also do not show clear separation. Species delimitation analyses based on COI sequence data, on the other hand, suggest five distinct genetic species. Despite the lack of diagnostic characters for these suggested species, the lineages can be classified at least into two morphological groups, the *bermudensis* and the *mangrovi* group which can only be distinguished by the arrangement of cuticular ventral carinae. Specimens within a group show nearly identical phenotypes, impeding morphological identification and hence rendering the found diversity cryptic. Stabilizing selection caused by the extreme conditions of the intertidal environment is suggested to be responsible for the found morphological stasis. The genetic lineages show more or less clear geographic patterns; in *C. mangrovi*, there is a northern, an Antillean, and a Pacific lineage, whereas in *C. bermudensis*, there is a Bermudian and a Caribbean lineage. In a few places, e.g., the Bahamas and Panama, distributions may overlap. Neither the found biogeographic pattern nor the observed ecological needs could explain the reason for the genetic diversification of Caribbean *Carinozetes*.

## Introduction

Oribatid mites are in large part typical terrestrial arthropods dwelling in habitats like soil, litter, or trees. Very few are associated with marine coastal habitats, as for example mangrove forests, boulder beaches, or rocky cliffs. The family Selenoribatidae represents one of the few marine-associated mite groups and presently includes nine genera, *Arotrobates* (Luxton 1992), *Carinozetes* (Pfingstl and Schuster 2012), *Indopacifica* (Pfingstl et al. 2019a, b), *Psednobates* (Luxton 1992), *Rhizophobates* (Karasawa and Aoki 2005), *Schusteria* (Grandjean 1968), *Selenoribates* (Strenzke 1961), *Thalassozetes* (Schuster 1963), and *Thasecazetes* (Pfingstl et al. 2017), with 29 species. Although the distribution of this family spans the globe, they are confined to subtropical and tropical shores, where they live exclusively in the intertidal zone between low and high tide (Pfingstl 2017). They basically feed on intertidal algae (Pfingstl 2013a) and can tolerate daily tidal submergence by using an elaborate plastron system allowing underwater respiration (Pfingstl and Krisper 2014). Most members of Selenoribatidae are known to occur in the Indo-Pacific region (Pfingstl and Schuster 2014), but recent studies (Pfingstl and Schuster 2012; Pfingstl 2013b, c; Pfingstl et al. 2016, 2017, 2019a) demonstrated that the diversity of these mites shows also high levels in other geographic regions, as for example the Western Atlantic and the Caribbean.

The Caribbean area shows a long and complex geological history, characterized by continental islands which broke off from mainland, land-bridge islands that were connected to the continent, uplifted limestone, and volcanic islands (Iturralde-Vinent 2006), and hence represents a focal area for evolutionary biologists. Presently, four genera of oribatid mites, *Carinozetes*, *Schusteria*, *Thalassozetes*, and *Thasecazetes*, are reported from this region (Pfingstl 2013c; Pfingstl and Lienhard 2017; Pfingstl et al. 2016, 2017, 2019a) whereas only *Carinozetes* and *Thasecazetes* have their biogeographic center in this region. *Thasecazetes* represents a recently discovered monotypic genus only known from the Lesser Antillean Island of Bonaire (Pfingstl et al. 2017). *Carinozetes*, on the other hand, was established when two new species, *Carinozetes trifoveatus* and *Carinozetes bermudensis*, were found and described from Bermuda (Pfingstl and Schuster 2012). Soon later, a comprehensive study (Pfingstl et al. 2014) revealed that the allegedly euryoecious *C. bermudensis* represented two different species that look nearly identical but have adapted to different ecological niches within the intertidal habitat. The true *C. bermudensis* occupies intertidal algae growing on a rocky substrate while the second formerly hidden species, namely *Carinozetes mangrovi*, dwells exclusively in intertidal algae growing on mangrove roots. Cryptic species are defined as taxa that are classified as a single nominal species because they are at least superficially anatomically identical (Bickford et al. 2007), and initially, this was also the case in *C. bermudensis*. The abovementioned study (Pfingstl et al. 2014), however, showed that the configuration of the ventral name giving cuticular carinae conspicuously differs between *C. bermudensis* and *C. mangrovi*, and hence represents a clear diagnostic trait that eliminates the cryptic nature of the species by allowing to differentiate between them.

*Carinozetes mangrovi* was subsequently found at various locations in the Caribbean, i.e., Jamaica, Barbados (Pfingstl and Schuster 2014), and the Dominican Republic (Pfingstl et al. 2016). Recent comprehensive sampling activities revealed further yet unpublished records of this species and of *C. bermudensis* from Central America and the Greater and Lesser Antilles indicating wide trans-Caribbean distribution areas for both species. However, Caribbean biota are known to show high levels of endemism and only a minority is represented by widespread species, presumably taxa with excellent dispersal abilities (Dziki et al. 2015). *Carinozetes* mites are tiny flightless arthropods and thus most likely not very good dispersers; hence, this contrasts with the observed large distribution areas. Due to this theoretical discrepancy, we analyzed COI sequence data from different populations of both species and this analysis revealed more than two clearly separate *Carinozetes* lineages to be present in the Caribbean. So, again, there is an indication of the existence of cryptic diversity within the genus *Carinozetes*.

In order to solve this enigma, the present paper provides (1) various analyses using the COI sequence data to confirm or dismiss the genetic lineages, (2) comprehensive morphometric data to test if found lineages can also be distinguished based on morphological criteria, and (3) a comparison of the distribution and ecology of each found lineage to test for diverging patterns.

## Material and methods

### Sample collection and locations

Samples of intertidal algae were scraped off the substrate (e.g., rock, mud, mangrove roots) with a knife and put in a Berlese-Tullgren funnel for approx. 24 h to extract the mites. Specimens were then stored in absolute ethanol for transport and further investigation. Sample locations and their details are given in Table 1.

**Table 1:** List of sample locations for each species, including sample code and collecting date

Species	Location	Country	Code	Date
*C. bermudensis*	Burnt Point Fort	Bermuda	BE_03	5 Aug 2011
	Soldier Bay*	Bermuda	BE_120	24 Apr 2012
	North of Lac Bay^1^	Bonaire	BON_88	23 Apr 1988
	Lac Bay*^1^	Bonaire	BO_01	24 Apr 2016
	Isla Colon, North coast*	Panama	PA_39	7 Feb 2017
	South Beach*	Bahamas	BH_16	20 Feb 2017
	Manzanillo^2^	Costa Rica	CR_01	12 Feb 2018
*C. mangrovi*	Hungry Bay	Bermuda	BE_128	2 May 2012
	New Providence Isl., South Beach	Bahamas	BH_15	20 Feb 2017
	Key Biscayne	FL, USA	FL_03-04	12 Feb 2017
	Florida Keys, Indian Key Fill	FL, USA	FL_10	13 Feb 2017
	Florida Keys, Islamorada	FL, USA	FL_17	13 Feb 2017
	West Palm Beach	FL, USA	FL_22	14 Feb 2017
	Naples, Lowdermilk Park	FL, USA	FL_26	16 Feb 2017
	Naples, Bonita Springs	FL, USA	FL_27-29	16 Feb 2017
	Pan. City, Mirador del Pacifique	Panama	PA_01	1 Feb 2017
	Pan. City, Plaza Quinto Centanario	Panama	PA_03	1 Feb 2017
	Pan. City, Escuela Republica de Mexico	Panama	PA_04	1 Feb 2017
	Pan. City, Punta Paitilla	Panama	PA_07	1 Feb 2017
	Punta Chamé	Panama	PA_13	3 Feb 2017
	Playa el Rompio*	Panama	PA_24	4 Feb 2017
	Discovery Bay	Jamaica	JA_04	19 Aug 2012
	Boca Chica*	Dominican Republic	DR_03	8 Feb 2016
	Samaná*	Dominican Republic	DR_10	11 Feb 2016
	Isla Magueyes	Puerto Rico	PR_05	14 Feb 2016
	Bois Jolan	Guadeloupe	GU_14	20 Feb 2016
	Trinité*	Martinique	MA_08	24 Feb 2016
	La Sagesse Beach	Grenada	GR_10	27 Feb 2016
	Cativá	Panama	PA_10	2 Feb 2017
	Isla Colon, STRI Institute	Panama	PA_33	7 Feb 2017
	Isla Colon, Boca del Drago	Panama	PA_45	8 Feb 2017
	New Providence Isl., South Beach	Bahamas	BH_13	20 Feb 2017
	South Beach*	Bahamas	BH_16	20 Feb 2017
	New Providence Isl., Montagu Beach	Bahamas	BH_23	22 Feb 2017

*Samples only used for morphological or molecular genetic analyses, not for morphometric analyses

^1^Samples collected by H. Schatz

^2^Samples collected by G. Kunz

### Genetic analyses

In total, 30 specimens of Caribbean *Carinozetes* spp. were analyzed. Total genomic DNA was extracted from single individuals preserved in absolute ethanol. Extraction was carried out using the Chelex method (Casquet et al. 2012) with some adjustments for small arthropods (whole specimens were crushed against the tube wall in microcentrifuge tubes containing 55 μl of a 10% Chelex solution with 2 μl Proteinase K). Samples were extracted for 3–4 h at 56 °C. A 564-bp fragment of the mitochondrial *cytochrome c oxidase subunit 1* gene (*COI*) was amplified using the primer pairs Mite COI-2F and Mite COI-2R (Otto and Wilson 2001). PCR conditions for the *COI* gene fragment are given in Pfingstl et al. (2014). DNA purification (with the enzyme cleaner ExoSAP-IT, Affymetrix; and the Sephadex G-50 resin, GE Healthcare) and sequencing steps (using the BigDye Sequence Terminator v3.1 Cycle Sequencing Kit, Applied Biosystems) were conducted after the methods published by Schäffer et al. (2008). Sequencing was performed in both directions on an automated capillary sequencer (ABI PRISM 3130xl, Applied Biosystems). Alignments were generated by means of the program MEGA6 (Tamura et al. 2013), as there were no gaps in the sequences alignments made by hand. Bayesian 50% majority-rule consensus tree was generated by means of MrBAYES 3.2.6 (Ronquist et al. 2012) applying an MC^3^ simulation with 20 million generations (10 chains, 2 independent runs, 10% burn-in, GTR+I+G model). Results were analyzed in TRACER v.1.6 (Rambaut and Drummond 2007) to check for convergence and to ensure the stationarity of all parameters. Neighbor-joining (NJ) tree was generated with MEGA6 (10,000 bootstrap replicates), and maximum likelihood (ML) analyses were carried out using RAxML (Stamatakis 2014) applying 10,000 bootstrap replicates and the GTR+gamma model. Distances based on the Kimura 2-parameter model (K2P) were calculated in MEGA6.

Molecular species delimitation was performed using three different methods. The Automatic Barcode Gap Discovery (ABGD, Puillandre et al. 2012) was conducted with default settings (simple distance) via the ABGD web server (http://wwwabi.snv.jussieu.fr/public/abgd/abgdweb.html). ABGD is an automatic procedure that sorts the sequences into putative species based on distance. The ABGD is a non-tree-based method and only requires an alignment file. In addition, two-tree-based species delimitation methods namely the general mixed Yule coalescent model (GMYC, Pons et al. 2006) and the multi-rate Poisson tree processes (mPTP, Kapli et al. 2017) were applied. GMYC was conducted by means of the splits package as implemented in R version 3.3.2 (R Core Team 2013) For GMYC analyses, ultrametric input trees are required. Therefore, BEAST 2 version 2.5.1 (Bouckaert et al. 2014) was used to generate a posterior sample of ultrametric trees. At first, the best-fitting substitution models (GTR+I) was selected by the smart model selection in PhyML (http://www.atgc-montpellier.fr/sms/, Lefort et al. 2017). Input file was constructed using BEAUti as implemented in BEAST applying a Yule tree model, a relaxed clock (Drummond et al. 2006; divergence rate of 2.15% cf. Salomone et al. 2002, Heethoff et al. 2007), and 50 million generations, resulting in 5000 trees (of which 10% were discarded as burn-in). TRACER v.1.6 was again used to verify the chains had reached stationarity. The 4500 post-burn-in trees were combined with TreeAnnotator (also implemented in the BEAST package). A single threshold was employed due to its better performance in delimitation (Fujisawa and Barraclough 2013). For the mPTP analysis, an input tree (with branch length) in a Newick format (from the BI analysis) was submitted to the mPTP web server (https://mptp.h-its.org/#/tree). The multi-rate Poisson tree processes method was selected.

All sequences obtained from this study were deposited in GenBank (www.ncbi.nlm.nih.gov/genbank; accession numbers [MK507820- MK507829]). Moreover, already published sequences of *C. bermudensis* and *C. mangrovi* (KF305231-KF305250, Pfingstl et al. 2014) were integrated into the alignment.

### Morphological analyses

For microscopic investigation in transmitted light, preserved animals were embedded in Berlese mountant. Drawings were made with an Olympus BH-2 Microscope equipped with a drawing attachment. These drawings were digitally remastered with the free and open-source vector graphics editor Inkscape (freeware available under www.inkscape.org).

For photographic documentation, specimens were air-dried and photographed with a Keyence VHX-5000 digital microscope.

Morphological terminology used in this paper follows that of Grandjean (1968) and Norton and Behan-Pelletier (2009).

### Morphometric analyses

**Fig. 1:**
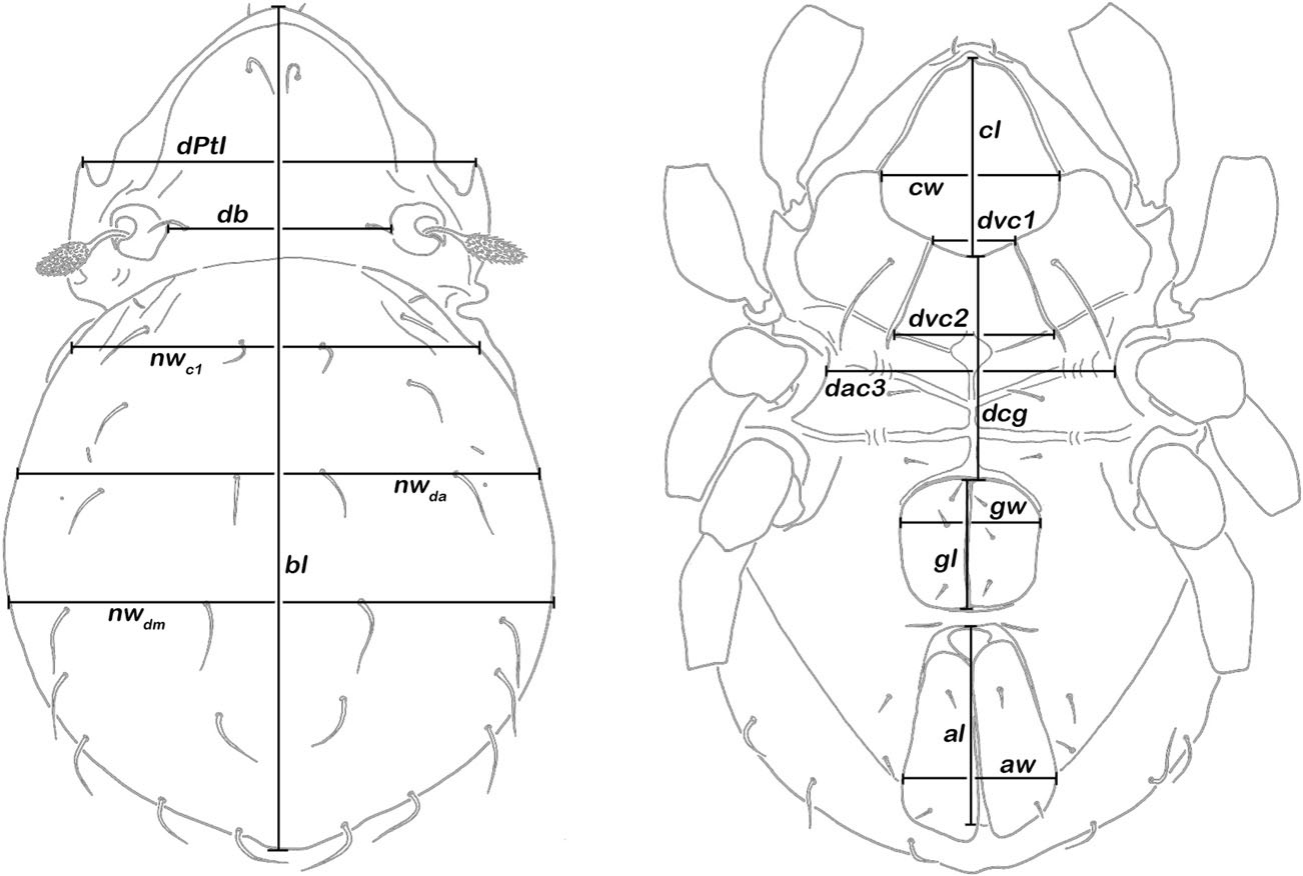
Graphic illustration of measured continuous variables shown on a simplified drawing of *Carinozetes mangrovi*. Left, dorsal aspect. *dPtI* distance between pedotecta 1, *db* distance between bothridia, *bl* body length, *nw*_*c1*_ notogastral width on level of seta *c*_*1*_, *nw*_*da*_ notogaster width on level of seta *da*, *nw*_*dm*_ notogastral width on level of seta *dm*. Right, ventral aspect. *cl* camerostome length, *cw* camerostome width, *dvc1* distance between anterior edges of ventral carinae, *dvc2* distance between posterior edges of ventral carinae, *dcg* distance between camerostome and genital orifice, *dac3* distance between acetabula 3, *gl* genital orifice length, *gw* genital orifice width, *al* anal orifice length, *aw* anal orifice width

Specimens were embedded in lactic acid for temporary slides, and measurements were done using a compound light microscope (Olympus BH-2) and ocular micrometer. A total of 16 continuous variables (Fig. 1) were measured in 433 *Carinozetes* specimens from 25 populations from various Caribbean locations (Central America, Greater Antilles, Lesser Antilles, Bahamas, North America, and Bermuda; for details refer to Table 1).

Multivariate analyses were conducted to reveal and assess morphometric differences between *Carinozetes* species and to investigate intraspecific variation across distribution areas.

Differences between *Carinozetes* populations were studied by canonical variates analysis (CVA), which was performed on ln(*x* + 1) transformed size-corrected data. Size correction was performed as described in Pfingstl et al. (2017). As preliminary investigations revealed strong sexual dimorphism, mainly in correlation with length and width of the genital opening, the two sexes were analyzed separately. Permutational multivariate analysis of variance (PERMANOVA) was conducted on all species and *post hoc* also in pairwise comparisons for testing the equality of means of the species. The performance of the classification by CVA was tested by calculating the number of specimens correctly classified by all-samples CVA and leave-one-out cross-validation CVA.

Intraspecific variation was investigated in all lineages except for Bermudian *C. bermudensis* because results for this lineage were already published in Pfingstl et al. (2014). Both sexes were analyzed together, but the variables *gl* and *gw*, which explain length and width of the genital opening, were excluded in order to minimize the effects of the sexual dimorphism. CVAs were performed on ln(*x* + 1) transformed raw and size-corrected data. The populations of *C. mangrovi* from diverse locations in Florida were pooled for this analysis. As only two populations for the Panamanian lineage were available and the results of the CVA could thus not be depicted in the form of a scatter plot, a principal component analysis (PCA) was performed. PERMANOVA was conducted for all lineages and the performance of the classification by CVA was evaluated like described above.

All analyses were performed with PAST 3.11 (Hammer et al. 2001).

## Results

### Genetic analyses

**Fig. 2:**
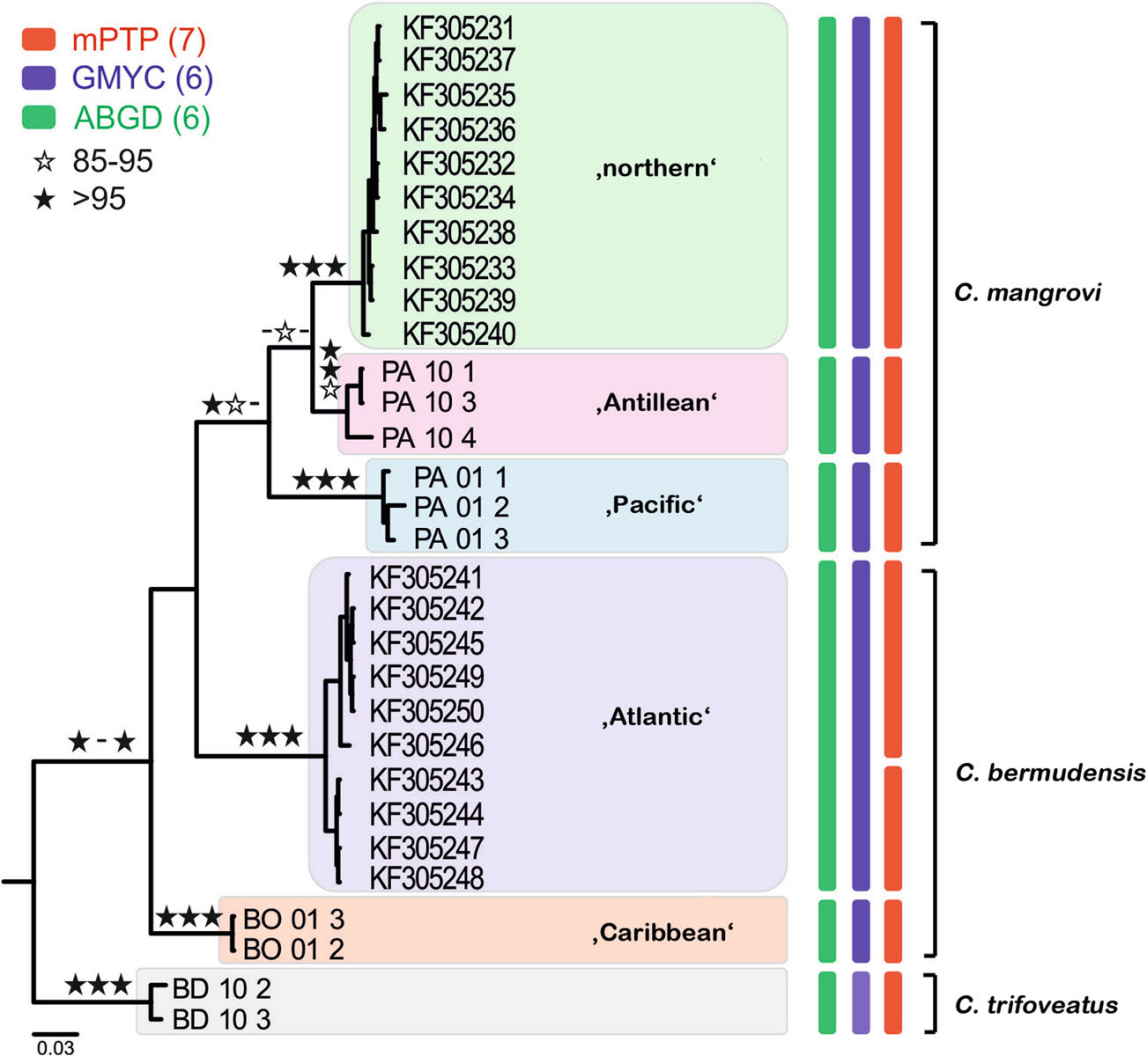
Bayesian inference tree based on COI sequences (564 bp) with the summary from all species delimitation analyses. Posterior probabilities (BI) and bootstrap values (NJ, ML) ranging from 85 to 95 are indicated by empty stars, values over 95 by black stars near nodes. Vertical bars at terminal branches specify delimited species obtained from three different approaches

**Fig. 3:**
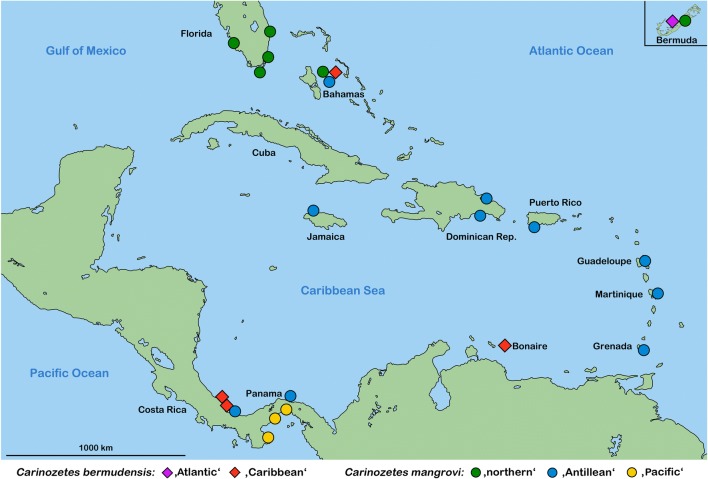
Map of the Caribbean showing the distribution of cryptic *Carinozetes* lineages. Circles represent lineages of the “*mangrovi* group” and squares refer to members of the “*bermudensis* group.” Small insert highlights the occurrence on Bermuda in the Western Atlantic

Bayesian inference (BI), maximum likelihood (ML), and neighbor-joining (NJ) analyses produced largely congruent phylogenies for all clades. The GMYC species delimitation analysis detected six putative species with high Yule support values (> 67) and the ABGD (partition 5–7) found also six species (*p* = 0.007743–0.021544); hence, both analyses classified the abovementioned clades as separate species (Fig. 2). These suggested species will be referred to as lineages in the following text, namely lineage “northern,” “Antillean,” “Pacific” (all *C. mangrovi*), and “atlantic,” “Caribbean” (both *C. bermudensis*) (Fig. 3). The mPTP analysis resulted in seven species in the data set, whereas the two branches of the *C. bermudensis* clade were supported as different species.

Genetic divergence (K2P distances) between lineages species respectively was basically high ranging from 7 to 20% whereas divergence within lineages was low ranging only from 1 to 2% (Table 2). Within the cryptic complex (*C. trifoveatus* excluded), the lowest mean genetic distances were detected between the “northern” and the “Antillean” *C. mangrovi* with 7%, whereas the highest distances were shown between the “atlantic” and the “Caribbean” *C. bermudensis* with 15% divergence.

**Table 2:** Mean K2P distances (Kimura 2-parameter model) within (given in italics) and between *Carinozetes* lineages

	*C. bermudensis* “Atlantic”	*C. bermudensis* “Caribbean”	*C. mangrovi* “northern”	*C. mangrovi* “Antillean”	*C. mangrovi* “Pacific”	*C. trifoveatus*
“Atlantic”	*0.01*					
“Caribbean”	0.12	*0.01*				
“northern”	0.13	0.13	*0.00*			
“Antillean”	0.16	0.12	0.07	*0.02*		
“Pacific”	0.15	0.13	0.12	0.11	*0.01*	
*C. trifoveatus*	0.19	0.18	0.18	0.18	0.20	*0.02*

### Morphology

**Fig. 4:**
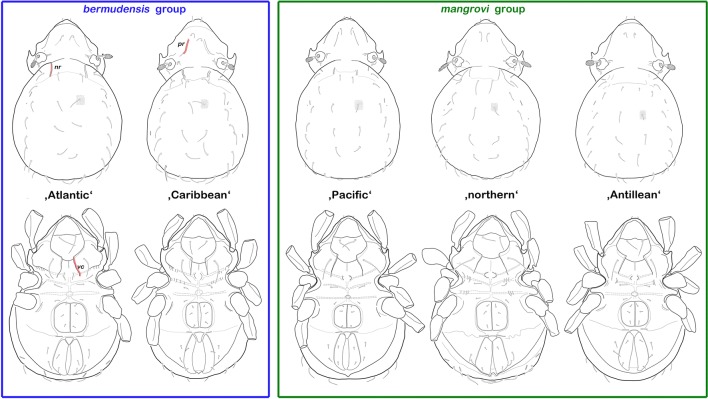
Graphic comparison of all Caribbean *Carinozetes* lineages highlighting the remarkable morphological similarity between and within the cryptic groups. Upper row, dorsal view; lower row, ventral view (distal leg segments omitted). Diverging traits exemplarily marked red; *pr* prodorsal ridge, *nr* notogastral ridge, and *vc* ventral carina

**Fig. 5:**
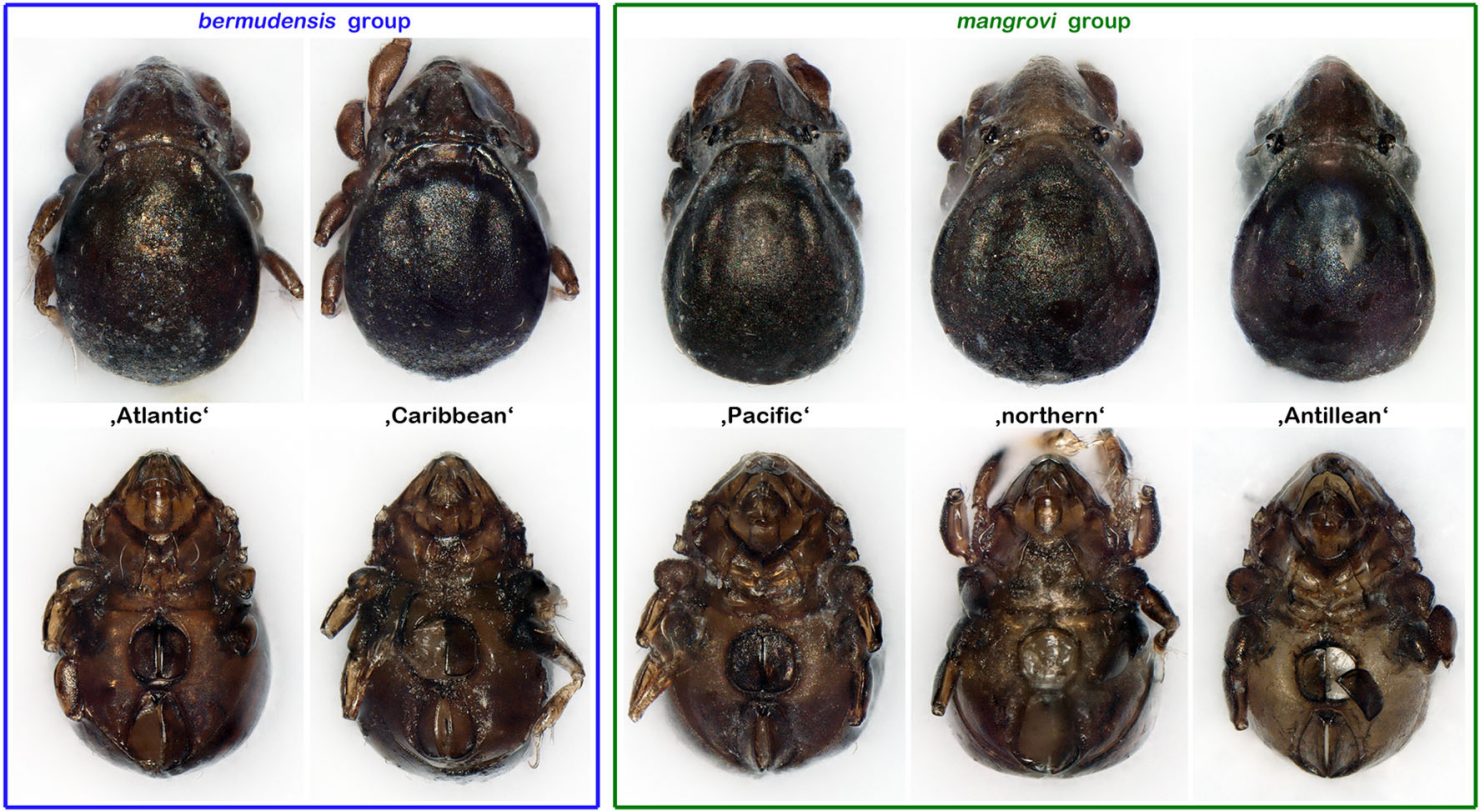
Photographic comparison (stacked stereomicroscopic images) of all Caribbean *Carinozetes* lineages highlighting the remarkable morphological similarity between and within the cryptic groups. Upper row, dorsal view; lower row, ventral view (legs were partly removed for better visibility of epimeral structures)

Basically, all genetic lineages look very similar and determination is only possible to a limited extent. Based on the configuration of ventral carinae, two morphological groups can be clearly distinguished: the first group, in which the ventral carinae are aligned in parallel, will be referred to as the “*bermudensis* group” and includes the original *C. bermudensis* (atlantic) and the “Caribbean” lineage; the second group, herein called the “*mangrovi* group,” consists of the “northern,” the “Antillean,” and the “Pacific” lineage and shows strongly converging ventral carinae (Figs. 4 and 5). Members of the “Caribbean” *C. bermudensis* possess conspicuous prodorsal and notogastral ridges whereas this trait is more or less weakly developed in the “Atlantic” *C. bermudensis* and all *C. mangrovi* lineages, and therefore, this genetic clade may be distinguished from all others, at least by the trained eye. Within the “*mangrovi* group,” specimens are hardly diverging, and hence, a clear distinction is unfeasible. The “Pacific” *C. mangrovi* shows faint prodorsal ridges, in contrast to the other two lineages, but this trait varies within the lineage and hence cannot be used as differentiating character. Members of the “northern” and the “Antillean” *C. mangrovi* are more or less completely identical in terms of morphology.

### Morphometry

#### Morphometric comparison/clades

Univariate statistics (Table 3) showed in all variables that at least one of the five clades of *Carinozetes* was significantly different from the others. In pairwise comparisons between the species, *dvc2* (distance between posterior edges of ventral carinae) was the variable that was significantly different in the highest number of possible combinations: it differed significantly between all lineages except between the “Atlantic” and the “Caribbean” *C. bermudensis*. Variable *dvc2* is also the only one that can be used as a discrete character for defining the two species groups, as was further demonstrated by CVA.

**Table 3:** Univariate statistics for five *Carinozetes* lineages

	*C. bermudensis* (*n* = 20) “Atlantic”					*C. mangrovi* (*n* = 187) “northern”					*C. bermudensis* (*n* = 33) “Caribbean”					*C. mangrovi* (*n* = 100) “Pacific”					*C. mangrovi* (*n* = 93) “Antillean”					KW	MWU
	min	max	*x*	sd	cv	min	max	*x*	sd	cv	min	max	*x*	sd	cv	min	max	*x*	sd	cv	min	max	*x*	sd	cv		
*bl*	334	385	*359*	12.36	0.03	331	388	*353*	11.54	0.03	313	385	*349*	20.05	0.06	319	381	*350*	12.78	0.04	319	382	*348*	12.32	0.04	**	e*
*dPtI*	154	169	*164*	4.06	0.02	157	175	*163*	3.81	0.02	148	172	*161*	7.85	0.05	148	169	*162*	4.21	0.03	151	182	*163*	5.34	0.03	*	
*db*	86	96	*90*	2.71	0.03	86	105	*92*	2.95	0.03	77	102	*88*	7.07	0.08	77	100	*90*	3.49	0.04	82	105	*92*	4.86	0.05	***	d*; b**; c***
*nwc1*	135	169	*152*	9.96	0.07	123	175	*152*	9.55	0.06	126	166	*144*	10.28	0.07	132	172	*151*	8.88	0.06	126	166	*148*	9.60	0.07	***	a, h*; b**
*nwda*	209	237	*222*	8.62	0.04	194	249	*221*	10.74	0.05	191	246	*214*	16.40	0.08	185	237	*218*	11.38	0.05	200	240	*218*	10.21	0.05	**	a*
*nwdm*	215	243	*233*	7.04	0.03	215	265	*236*	9.36	0.04	203	249	*224*	13.69	0.06	203	252	*228*	10.14	0.04	206	259	*229*	10.34	0.05	***	a, b. c***
*cl*	92	102	*96*	2.63	0.03	86	102	*94*	2.93	0.03	86	105	*96*	5.62	0.06	86	102	*93*	3.19	0.03	86	102	*94*	3.61	0.04	***	c, e*; d, h**
*cw*	74	80	*75*	2.03	0.03	74	80	*77*	1.80	0.02	71	83	*75*	3.76	0.05	71	83	*77*	1.98	0.03	71	86	*78*	2.80	0.04	***	f***
*dvc1*	22	31	*28*	2.39	0.09	24	41	*36*	2.62	0.07	22	43	*28*	4.23	0.15	34	43	*38*	1.90	0.05	31	46	*38*	2.87	0.08	***	f, i*; d, e**
*dvc2*	34	49	*42*	4.10	0.10	62	83	*70*	3.53	0.05	34	71	*44*	6.71	0.15	65	82	*74*	3.18	0.04	55	77	*68*	4.63	0.07	***	a*; b, c, d, e, f, h, i, j***
*efw*	15	22	*18*	2.05	0.11	12	22	*17*	2.06	0.12	12	22	*16*	2.40	0.15	12	20	*17*	2.03	0.12	12	25	*17*	2.43	0.15	**	e, f*; d, g**
*dcg*	77	95	*83*	4.82	0.06	71	89	*81*	3.46	0.04	66	95	*78*	6.48	0.08	74	93	*85*	3.75	0.04	68	95	*79*	4.95	0.06	***	c, g*; b**
*dac3*	108	120	*114*	3.41	0.03	99	123	*113*	3.51	0.03	105	129	*117*	5.78	0.05	108	123	*117*	3.34	0.03	105	126	*113*	4.92	0.04	***	i**; b, c, j***
*gl*	52	68	*62*	5.67	0.09	49	71	*59*	5.43	0.09	47	68	*58*	6.34	0.11	46	68	*58*	6.52	0.11	46	71	*58*	5.51	0.09	*	
*gw*	53	71	*62*	6.10	0.10	57	77	*66*	4.93	0.08	49	71	*61*	6.55	0.11	52	74	*64*	6.24	0.10	55	74	*63*	5.32	0.08	***	a, b**
*al*	83	92	*86*	3.00	0.03	77	92	*86*	2.69	0.03	74	92	*81*	4.59	0.06	74	89	*83*	3.11	0.04	74	92	*82*	4.03	0.05	***	e, f, g**; a, b, c***
*aw*	59	71	*64*	2.80	0.04	59	72	*66*	2.62	0.04	55	68	*62*	4.31	0.07	54	66	*60*	2.76	0.05	55	77	*63*	3.97	0.06	***	d*; a, b, c***

Univariate statistics for five *Carinozetes* lineages showing significant differences between the lineages in each measured variable

*min* minimum, *max* maximum, *x* mean, *sd* standard deviation, and *cv* coefficient of variation

Results of Kruskal–Wallis (KW) and Mann–Whitney *U* (MWU) tests are given

*p < 0.05

**p < 0.01

***p < 0.001

a “northern” vs. “Caribbean”; b “northern” vs. “Antillean”; c “northern“ vs. “Pacific“; d “northern” vs. “Atlantic”; e “Atlantic” vs. “Antillean”; f “Atlantic” vs. “Pacific”; g “Atlantic” vs. “Caribbean”; h “Caribbean” vs. “Pacific”; i “Caribbean” vs. “Antillean”; j “Antillean” vs. “Pacific”

**Fig. 6:**
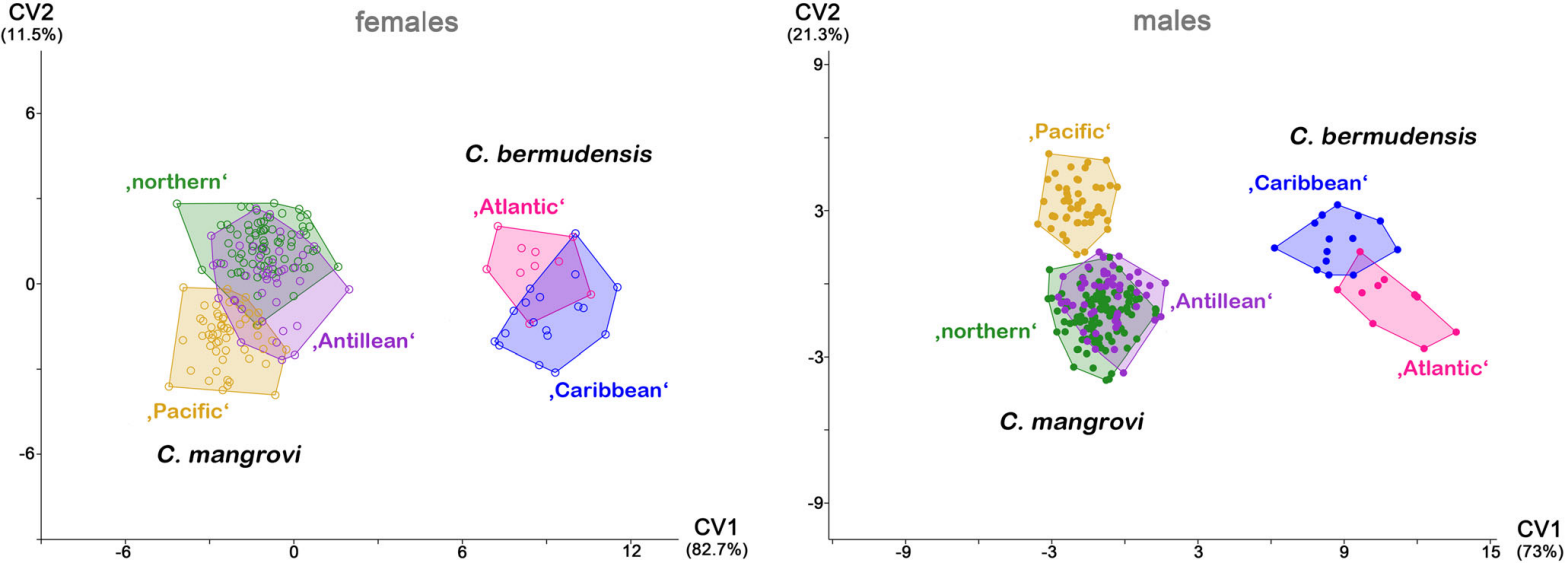
CVA scatter plots of five *Carinozetes* lineages; female and male specimens shown in separate graphs. Percentage of total variation explained by the axes given in parentheses

CVA conducted on both males and females clearly showed two species groups separated on CV1, one consisting of the *C. bermudensis* lineages and the other of the *C. mangrovi* lineages (Fig. 6). Variable *dvc2*, in coincidence with the results of the univariate statistic, was always most responsible for the separation between the two groups (Table 4). The differences between clades within the respective species groups were always more pronounced in the males. In the “*bermudensis* group,” the females of the “Atlantic” and the “Caribbean” lineage considerably overlapped in CVA, while the males formed two clearly separated clusters in the respective analysis. In the “*mangrovi* group,” females of the “northern” and the “Pacific” lineage were separated along CV2 in CVA, and the “Antillean” lineage overlapped with both. The males of the “northern” and the “Pacific” *C. mangrovi* were also separated along CV2, and here, the “Antillean” *C. mangrovi* overlapped only with the “northern” lineage. In both sexes, the variables contributing most to separation on CV2 were *aw*, *dac3* (distance between acetabula 3), and *dcg* (distance between camerostome and genital orifice).

**Table 4:** Loadings of the two canonical axes CV1 and CV2 for CVA on five *Carinozetes* lineages. High loadings explaining differences between species are given in italics

	Females		Males		Females		Males	
	Raw data				Size-corrected data			
	CV1	CV2	CV1	CV2	CV1	CV2	CV1	CV2
*bl*	0.000	− 0.005	0.000	0.002	0.005	− 0.001	0.004	0.002
*dPtI*	0.000	− 0.004	0.001	0.003	0.003	− 0.001	0.003	0.001
*db*	0.002	− 0.008	0.003	0.008	0.002	0.001	0.001	− 0.002
*nwc*_*1*_	0.003	− 0.002	0.001	0.004	0.001	− 0.002	0.003	0.000
*nwda*	0.001	− 0.008	0.001	0.006	0.003	0.001	0.003	− 0.001
*nwdm*	0.002	− 0.012	0.002	0.010	0.003	0.004	0.002	− 0.004
*cl*	− 0.003	− 0.005	− 0.003	0.001	0.005	− 0.001	0.004	0.002
*cw*	0.002	− 0.002	0.002	0.002	0.002	− 0.002	0.002	0.001
*dvc1*	0.028	0.008	0.026	− 0.005	− 0.006	− 0.003	− 0.006	0.002
*dvc2*	*0*.*047*	0.003	*0*.*045*	− 0.003	*− 0*.*016*	− 0.003	*− 0*.*016*	0.003
*dcg*	0.004	0.008	0.001	− 0.011	0.000	*− 0*.*006*	0.002	*0*.*007*
*dac3*	− 0.001	0.009	− 0.001	− 0.007	0.004	*− 0*.*008*	0.004	*0*.*006*
*gl*	0.000	− 0.010	0.000	*0*.*018*	0.002	0.002	0.002	− 0.005
*gw*	0.004	− 0.009	0.007	*0*.*016*	0.001	0.002	− 0.001	− 0.005
*al*	0.001	− 0.014	0.001	0.007	0.002	0.004	0.002	− 0.001
*aw*	0.000	*− 0*.*027*	0.001	*0*.*019*	0.002	*0*.*009*	0.002	*− 0*.*006*

PERMANOVA on all five lineages and in pairwise comparisons always showed significant differences (*p* < 0.01) in both sexes.

The percentages of specimens correctly classified by CVA (all-samples CVA/leave-one-out cross-validated CVA) in females were 86.76/81.37% in raw data and 86.76/82.35% in size-corrected data, and in males 89.91/85.53% in raw data and 89.47/86.40% in size-corrected data.

#### Morphometric comparison/populations of a lineage

**Fig. 7:**
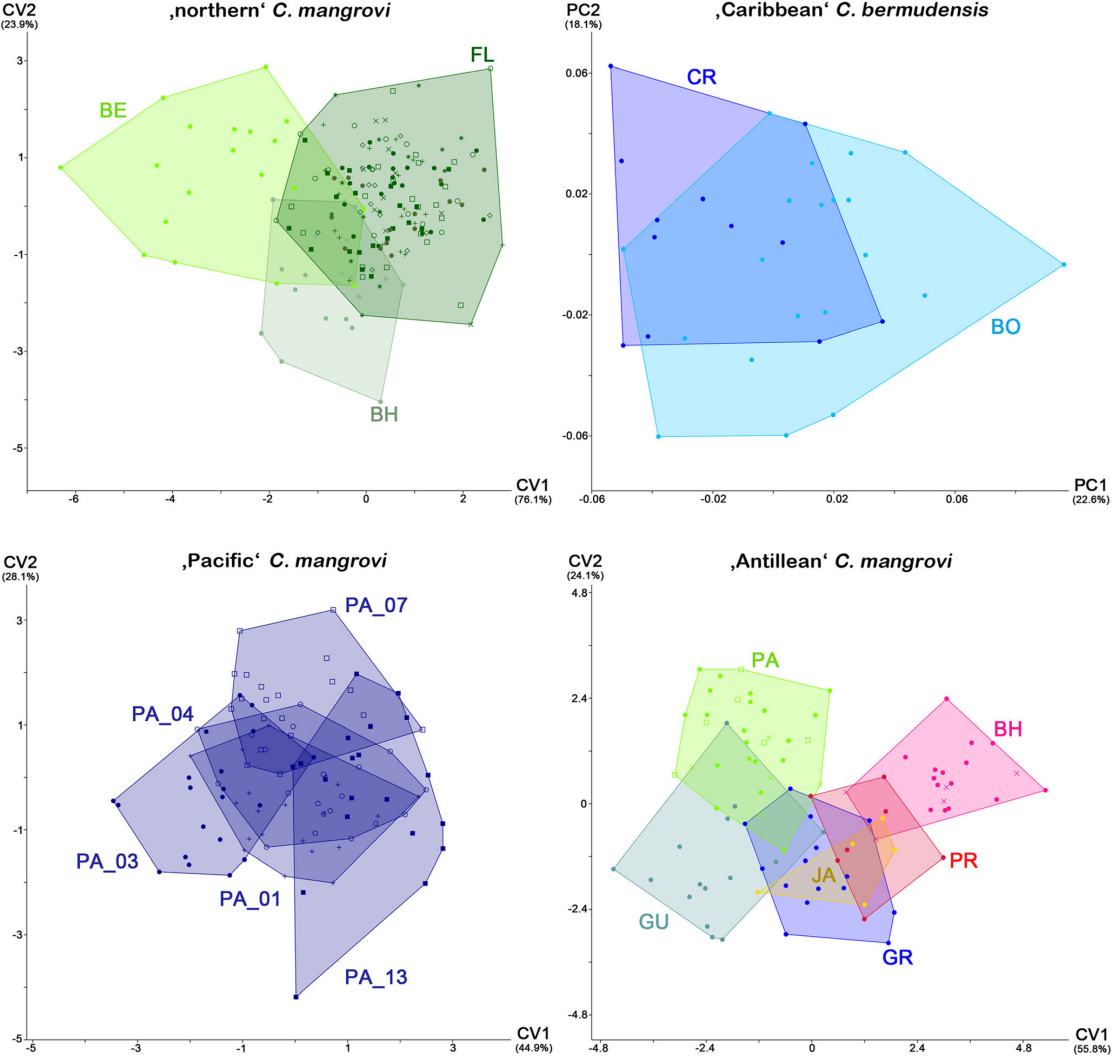
CVA scatter plots of different populations of three *Carinozetes mangrovi* lineages and PCA graph for two different populations of the “Caribbean” *C. bermudensis* on size-corrected data. Percentage of total variation explained by the axes given in parentheses. *Carinozetes mangrovi* specimens from different locations in Florida and individuals from Panama and Bahamas each pooled for analysis but different populations are still given as different symbols. BE, Bermuda; BH, Bahamas; BO, Bonaire; CR, Costa Rica; FL, Florida; GR, Grenada; GU, Guadeloupe; JA, Jamaica; and PA, Panama

The three populations of the “northern” *C. mangrovi* (Bahamas, Bermuda, and Florida) formed clear groups with small overlaps in CVA on both raw and size-corrected data (Fig. 7). PERMANOVA on all three populations revealed that at least one of them differed significantly (*p* < 0.01) from the others, and pairwise comparisons found significant differences between all populations except between the populations from Bahamas and Florida in raw data. All-samples CVA correctly classified 84.49% of the specimens in raw data and 83.96% in size-corrected data, and leave-one-out cross-validated CVA correctly classified 79.68% and 78.61% in raw and size-corrected data, respectively.

The “Caribbean” *C. bermudensis* was analyzed from Costa Rica and Bonaire, and these two populations were clearly separated in PCA on raw data but largely overlapped in PCA on size-corrected data, indicating that the found differences can mostly be explained by size (Fig. 7). PERMANOVA revealed that the two populations were highly significantly different in both raw and size-corrected data. The percentages of correctly classified specimens by CVA were 100% and 96.88% in all-samples CVA on raw and size-corrected data, respectively. The corresponding percentages in leave-one-out cross-validated CVA were 96.88% and 84.38%.

The five populations of the “Pacific” *C. mangrovi* from Panama largely overlapped in both CVAs (Fig. 7), but in the raw data, the population PA_03 (Panama City) was slightly separated from the other populations. PERMANOVA revealed that at least one of the five populations was highly significantly different from the others in both raw and size-corrected data. In pairwise comparisons, PA_01 (Panama City) and PA_03 (Panama City) were significantly different from PA_07 (Panama City) and PA_13 (Punta Chamé) in both raw data and size-corrected data. Additionally, PA_03 was significantly different from PA_04 (Panama City) but only in raw data. All-samples CVA correctly classified 75% in raw data and 63% in size-corrected data, and leave-one-out cross-validated CVA correctly classified 52% and 48% in raw and size-corrected data, respectively.

Both CVA on raw and size-corrected data separated the six populations of the “Antillean” *C. mangrovi* (from Bahamas, Grenada, Guadeloupe, Jamaica, Panama, and Puerto Rico), with some populations clearly overlapping (Fig. 7). PERMANOVA revealed highly significant differences at least between two populations in both raw and size-corrected data. Pairwise comparisons showed more significant differences between populations in size-corrected data. In raw data, the population from the Bahamas differed from those from Grenada, Guadeloupe, Jamaica, and Panama, the population from Jamaica also differed from Grenada and Guadeloupe, and the specimens from Grenada furthermore differed from those from Panama. In the size-corrected data, the population from the Bahamas was significantly different from all other populations, the population from Panama differed from those from Grenada, Guadeloupe, Jamaica, and Puerto Rico, and the population from Guadeloupe differed from those from Grenada, Jamaica, and Puerto Rico.

All-samples CVA correctly classified 89.25% of specimens in raw data and 88.17% in size-corrected data, and leave-one-out cross-validated CVA correctly classified 74.19% in raw and 73.12% in size-corrected data.

## Discussion

### Cryptic diversity

Among biologists, there are contrasting opinions concerning the nature of cryptic diversity, some researchers claim that cryptic species do not exist because they are nothing more than an incompatibility of species concepts (e.g., Heethoff 2018) while others are convinced to find evolutionary processes hidden in cryptic species (e.g., Struck et al. 2018a). Indeed, there are many different definitions of cryptic diversity (Bickford et al. 2007); consequently, inconsistencies in the use of these definitions may hamper to draw conclusions about the prevalence and implications of cryptic diversity (Struck et al. 2018a). It is also true that there are different partially incompatible species concepts (De Queiroz 2007) and that prioritizing one over the other may result in artificial cryptic diversity (Heethoff 2018). Nevertheless, the phenomenon of high phenotypic similarity despite restricted gene flow is real, as could be well demonstrated in diverse metazoan taxa (Struck et al. 2018b). Investigating these cases may allow us to better understand evolutionary processes, like parallelism, convergence, and stasis (Struck et al. 2018a). However, dealing with cryptic diversity requires using a species concept to determine species boundaries and this may lead to the aforementioned problems with incompatible concepts resulting in grouping artifacts (Heethoff 2018).

In the present case, separating morphological features are absent in the studied *Carinozetes* lineages; hence, the morphological species concept and the property of disparity is not applicable. *Carinozetes* species are sexually reproducing; therefore, testing reproductive isolation would theoretically be possible but practically cross-breeding experiments would last for years due to low reproductive rates and difficulties in simulating the intertidal environment in the lab. Nonetheless, the “northern” and the “Antillean” *C. mangrovi* as well as the “Caribbean” *C. bermudensis* were found to occur syntopically or in close vicinity on the Bahamas, and no morphologically or genetically intermediate types or hybrids could be detected. This points to reproductive isolation between the groups which would allow to apply the biological species concept, but of course, the final proof is lacking. Molecular genetic data, on the other hand, renders the taxa clearly as separately evolving metapopulation lineages and thus would confirm the species in the sense of De Queiroz (2007) who proposed a unified species concept. So, from a genetic point of view, these lineages may already represent different species, but presently, additional proof, as for example reproductive isolation or other separating non-morphological characteristics, is lacking. Therefore, we refrain from assigning species rank to the lineages until further proof is found.

However, the question arises why these lineages show high levels of genetic divergence while their morphology exhibits only subtle differences. Based on an estimated molecular divergence rate of 2.15% per million years for the *COI* gene of oribatid mites (Heethoff et al. 2007), the cryptic *Carinozetes* lineages radiated approx. 3 to 7 mya; therefore, recent diversification can be excluded as a possible cause for the cryptic appearance between and within groups. The common occurrence in the extreme intertidal environment, on the other hand, may have played a role because extreme habitat conditions might impose stabilizing selection resulting in highly conserved morphologies (Colborn et al. 2001; Lefébure et al. 2006; Bickford et al. 2007). The stabilizing selection was already suggested to be responsible for the cryptic appearance of *Litoribates floridae*, another Caribbean intertidal mite (Pfingstl et al. 2019a), and the same may apply to the Caribbean *Carinozetes*. They have diversified in the Caribbean and have colonized vast regions of this area, but all have been subject to the same extreme conditions of the littoral zone, like wave action and daily submergence, and this may have caused the observed morphological stasis.

### Population structure

Despite the morphological similarity between the lineages, populations of each lineage show variations as indicated by morphometric data. One might argue that morphological stasis should counteract intraspecific variation which is true to a large extent. But stabilizing selection affects only characters that are subject to the strong selective constraints present in the extreme intertidal environment. Other unaffected characters still may show some variation due to ecological differences or genetic drift. Most found variation concerns overall body size and differences in size are basically assumed to represent non-genetic intraspecific variation caused by environmental factors (Jungers et al. 1995). Moreover, the found variation was subtle and basically low and could only be detected by morphometric analyses. Therefore, this low variation does not affect morphological stasis.

Nevertheless, morphometry allowed to show slightly diverging morphologies between the populations of a single lineage. In the “Caribbean” *C. bermudensis*, the Bonaire individuals differ significantly from the Costa Rican specimens in overall size, as indicated by the large overlap in size-corrected data. Specimens from Bonaire are on average 30 μm larger than Costa Rican individuals. The intertidal mite *Fortuynia hawaiiensis* showed conspicuous overall size variation between populations from different islands of the archipelago, which was assumed to be a result of different microclimates (Pfingstl and Jagersbacher-Baumann 2016). The responsible factors in the present case are unknown, but specimens from Bonaire dwelled in mangroves whereas the Costa Rican population inhabited a rocky intertidal area. Algal food supply in a mangrove forest is probably more diverse and extensive than on rocky substrate, and this may result in larger body sizes. However, such size differences could not be found in the “Antillean” *C. mangrovi* although populations alternatively occurred in mangrove forests and rocky shore.

Basically, *C. mangrovi* populations do not show remarkable size variations but rather exhibit divergences in specific variables. Each lineage varies in other variables; some of the variables are at least part of the same body region. In the “northern” and the “Pacific” lineage, highest variations mainly concern body width, and in the “Antillean” populations, divergences can be found in ventral carinae and anal region. As there is no specific overall pattern in variance between populations, these differences may be results of genetic drift, but presently, this is just conjecture. Intraspecific variation correlated with the distance between the islands in the intertidal mite *Alismobates galapagoensis* from Galapagos (Pfingstl and Baumann 2017), but in the studied *Carinozetes* lineages, no such correlation could be detected.

### Biogeography

When *C. bermudensis* and *C. mangrovi* were discovered on Bermuda, they were supposed to originate from Caribbean populations that have colonized the archipelago via transport along ocean currents (Pfingstl et al. 2014). The present records clearly support this assumption as populations of both species are largely distributed in the Caribbean area.

The found genetic *Carinozetes* lineages show more or less clear geographic patterns; the “northern” *C. mangrovi* occurs in the northern Caribbean area, from Florida and the Bahamas to Bermuda in the western Atlantic. The populations from Florida lie exactly in the path of the Gulf Stream which flows northwards passing Bermuda, and hence, the Bermudian populations may be derived from these. The “Antillean” *C. mangrovi* lineage shows a trans-Caribbean distribution with some records from Central America, as well as the Bahamas, but most records are reported from the Greater and Lesser Antilles. Either this lineage originated from a widely distributed Pro-Antillean stock or it shows good dispersal and colonization abilities. Further molecular genetic data is necessary to answer this question. The “Pacific” *C. mangrovi* lineage was only found on pacific shorelines of Panama; this lineage was probably separated from the other Caribbean lineages when the Isthmus of Panama finally closed about 3 million years ago (e.g., Iturralde-Vinent 2006).

The “Caribbean” *C. bermudensis* shows a disjunct distribution with records from Panama, Costa Rica, Bonaire, and the Bahamas. The ancestor of this lineage may have had a wider continuous distribution but possibly got extinct or replaced by other *Carinozetes* lineages in several regions of the Caribbean. The “Atlantic” *C. bermudensis*, on the other hand, is presently restricted to the small archipelago of Bermuda.

Considering the two species as a whole, there is no specific biogeographic pattern indicating that vicariance is responsible for the lineage separation. In some places, as for example in Panama or on the Bahamas, members of both species even occur syntopically.

### Ecology

Upon their discovery on Bermuda, *C. bermudensis* and *C. mangrovi* were suggested to use different ecological niches within the intertidal environment (Pfingstl et al. 2014), i.e., *C. bermudensis* dwells in algae on a rocky substrate while *C. mangrovi* exclusively inhabits algae growing on mangroves. The present study does not support this assumption because the “Caribbean” *C. bermudensis* and the “Antillean” *C. mangrovi* occurred equally on rocky substrate and mangrove forests and hence show a wider ecological range. Apart from these two, the “Pacific” *C. mangrovi* was mainly found on rocks and the “northern” *C. mangrovi* occurred exclusively on mangrove roots covered with algae; hence, they may have preferences for these habitats.

Based on these observations, the ecological needs within the two morphological groups vary, and therefore, a correlation between ecology and the morphological group can be excluded.

## Conclusions

The present case of two very similar morphological species containing cryptic diversity indicates that phenotypic similarity caused by stabilizing selection may represent a common phenomenon in intertidal mites. Moreover, many littoral arthropods, e.g., collembolans, staphylinid beetles, and isopods, are subject to the same conditions and show similar dispersal abilities and therefore may also contain unexpected cryptic diversity. The present study also demonstrates that integrative approaches are needed to uncover hidden diversity and to identify and document their nature. The evolutionary history of the cryptic *Carinozetes* complex, resulting in different species and lineages with various distribution patterns and ecological needs, was surely shaped by a complex interplay of geological, dispersal, and extinction events, but understanding the exact underlying evolutionary processes requires further comprehensive research.

## Data Availability

The molecular genetic datasets generated during and/or analyzed during the current study are available in the GenBank repository, [https://www.ncbi.nlm.nih.gov/genbank/]. Morphometric data generated during and/or analyzed during the current study are available from the corresponding author on request.
